# MiR-146a-5p engineered hucMSC-derived extracellular vesicles attenuate *Dermatophagoides farinae*-induced allergic airway epithelial cell inflammation

**DOI:** 10.3389/fimmu.2024.1443166

**Published:** 2024-09-19

**Authors:** Jiaxi Liu, Zuyu Xu, Jinyan Yu, Xiao Zang, Shangde Jiang, Shuyue Xu, Wei Wang, Shanchao Hong

**Affiliations:** ^1^ School of Clinical Laboratory Medicine, Nanjing Medical University, Nanjing, Jiangsu, China; ^2^ School of Public Health, Nanjing Medical University, Nanjing, Jiangsu, China; ^3^ Department of Clinical Laboratory, Jiangnan University Medical Center, Wuxi, Jiangsu, China; ^4^ National Health Commission Key Laboratory on Parasitic Disease Prevention and Control, Jiangsu Provincial Key Laboratory on Parasites and Vector Control Technology, Jiangsu Institute of Parasitic Diseases, Wuxi, Jiangsu, China

**Keywords:** hucMSC-derived extracellular vesicles, MiR-146a-5p, *D. farinae*, allergic airway inflammation, NF-κB/NLRP3

## Abstract

**Introduction:**

Allergic asthma is prevalent in children, with Dermatophagoides farinae as a common indoor allergen. Current treatments for allergic airway inflammation are limited and carry risks. Mesenchymal stem cell-derived extracellular vesicles (MSC-EVs) show promise as a cell-free therapeutic approach. However, the use of engineered MSC-EVs for D. farinae-induced allergic airway epithelial cell inflammation remains unexplored.

**Methods:**

We generated miR-146a-5p-engineered EVs from human umbilical cord mesenchymal stem cells (hucMSCs) and established D. farinae-induced mouse and human bronchial epithelial cell allergic models. Levels of IL-1β, IL-18, IL-4, IL-5, IL-6, IL-10, IL-33, TNF-α and IgE were detected using ELISA. The relative TRAF6 and IRAK1 mRNA expression was quantified using qPCR assay and the NLRP3, NF-κB, IRAK1 and TRAF6 protein expression was determined using Western blotting. The regulatory effect of IRAK1 and TRAF6 by miR-146a-5p was examined using a dual luciferase reporter assay, and the nuclear translocation of NF-κB p65 into 16-HBE cells was evaluated using immunofluorescence assay.

**Results:**

Treatment with hucMSC-EVs effectively reduced allergic inflammation, while miR-146a-5p engineered hucMSC-EVs showed greater efficacy. The enhanced efficacy in alleviating allergic airway inflammation was attributed to the downregulation of IRAK1 and TRAF6 expression, facilitated by miR-146a-5p. This downregulation subsequently led to a decrease in NF-κB nuclear translocation, which in turn resulted in reduced activation of the NLRP3 inflammasome and diminished production of inflammatory cytokines, including IL-6, TNF-α, IL-1β and IL-18.

**Conclusion:**

Our study underscores the potential of miR-146a-5p engineered hucMSC-EVs as a cell-free therapeutic strategy for D. farinae-induced allergic airway inflammation, offering a promising avenue for boosting anti-inflammatory responses.

## Introduction

As a significant subtype of asthma, allergic asthma is commonly prevalent in children. It has a global prevalence of approximately 11% among children aged 6-7 years old and 14% among those aged 13-14 years old (1). The economic burden of childhood asthma is particularly significant in low-income and middle-income countries (2). In children with allergic asthma, the most prevalent indoor allergens globally are derived from *Dermatophagoides farinae* (*D. farinae*), the predominant species of house dust mites (HDMs) (3).

Inflammasome is a multi-protein complex composed of NLRP3, apoptosis speck-like protein containing a caspase recruitment domain (ASC) and precursor Caspase-1 (pro-Caspase-1). HDM activates airway epithelial cells via Toll-like receptor (TLR), leading to NF-κB activation and secretion of pro-inflammatory cytokines, such as IL-6 and IL-1β (4), and also activates NLRP3 to promote HDM-induced inflammatory response in allergic asthma (5, 6). The findings of various studies indicate that NLRP3 inhibitors, such as OLT1177^®^ can decrease NLRP3 activity, reduce the release of proinflammatory IL-1β, and alleviate pathophysiological features in mouse models of allergic asthma (7). Chrysin, an anti-inflammatory and anti-allergic drug, has been shown to inhibit NF-κB and suppress NLRP3 activation in an asthma model (8). The regulation of NLRP3 via NF-κB in other inflammatory diseases has also been extensively studied. For example, in mice with experimental colitis, the REV-ERBα clock gene can indirectly repress NLRP3 activation by reducing NF-κB p65 transcription (9). Therefore, in patients with allergic asthma, the TLR/NF-κB/NLRP3 signaling pathway plays a crucial role in regulating allergic airway epithelial inflammation.

For children with allergic asthma, bronchodilators as well as steroids can reduce airway inflammation, improve asthma symptoms, and reduce the risk of severe asthma exacerbations and death, which are the first-line therapeutic options. However, for some children who do not improve with treatment, other therapies such as allergen-specific immunotherapy (AIT) or biologic therapy are still needed (10). AIT is not recommended for severe uncontrolled asthma. While these therapies are primarily suitable for patients with known sensitization to specific allergens, they do come with certain risks (11). Hence, there is an urgent need for the development of more effective treatments aimed at reducing inflammation in patients with allergic asthma. Recent research has shown that mesenchymal stem cells (MSCs) can effectively inhibit not only airway inflammation but also airway remodeling in animal models of asthma (12). MSC-derived extracellular vesicles (MSC-EVs) are considered a promising cell-free therapeutic agent due to their low immunogenicity, convenient storage, and high biosafety (13). For instance, it has been demonstrated that MSC-EVs administration through the nasal route significantly reduces the allergic airway inflammation induced by ovalbumin (OVA) (14). It is evident that this research was conducted using the OVA sensitization model. *D. farinae* is recognized as one of the primary allergens contributing to allergic asthma in humans. However, there is still a lack of further investigation on utilizing engineered EVs for the improved treatment of *D. farinae*-sensitized allergic airway inflammation. Hence, our study aims to explore the potential benefits of MSC-EVs in addressing allergic airway inflammation specifically sensitized by *D. farinae*.

MSC-EVs can be loaded with therapeutic drugs, serving as a potential delivery system that combines the autoimmunomodulatory potential of the EVs with the therapeutic function of the loaded drug. MicroRNAs, such as miR-146a-5p, negatively regulate NF-κB activation and the NLRP3 inflammasome signaling pathway (15–17). MiR-146a/b has demonstrated anti-inflammatory effects by suppressing NF-κB signaling and inhibiting the production of pro-inflammatory chemokines (18). Another study showed that miR-146a-5p can reduce allergic airway inflammation by inhibiting NLRP3 inflammasome activation in macrophages (19). In a mouse model of eosinophilic airway inflammation dominated by ILC2 cells, small vesicles derived from human umbilical cord MSCs (hucMSCs) demonstrated a reduction in ILC2 cell levels, inflammatory cell infiltration, and mucus production. RNA-seq analysis confirmed the significant upregulation of miR-146a-5p in these vesicles, which mediated their therapeutic effects (20). However, the specific mechanisms underlying these effects have not been extensively elucidated. Airway epithelial cells play a crucial role in protecting against exposure to allergic allergens. Therefore, the aim of our study was to specifically investigate the potential of utilizing EVs derived from miR-146a-5p-engineered MSCs and explore whether it has better anti-inflammatory effects on allergic airway inflammation induced by *D. farinae* in airway epithelial cells. Additionally, we intend to further explore and gain insights into the underlying mechanisms involved in this process.

HucMSCs possess distinct advantages such as non-invasive harvesting, ethical compliance, superior proliferation capabilities, and enhanced anti-inflammatory properties compared to MSCs derived from other tissues. Our study focuses on exploring the therapeutic potential of engineered EVs from hucMSCs with elevated miR-146a-5p expression in a model of *D. farinae*-induced allergic airway inflammation. Additionally, we have preliminarily demonstrated that miR-146a-5p-engineered hucMSC-EVs mitigates allergic airway inflammation through the modulation of the NF-κB/NLRP3 pathway.

## Materials and methods

### Ethical statement

All experimental protocols were approved by the Medical Ethics Committee of Wuxi No.2 People’s Hospital. The approval document number was 2022-Y-119 for the use of human umbilical cord and collection of serum samples from children. The written informed consent was obtained from the participant following a detailed description of the purpose of the present study. Animal experiments were performed strictly following the 3R principle and international and national laws, regulations and guidelines for the care and management of laboratory animals and the approval document number 2022-Y-131 for animal experiment.

### Cell culture

HucMSCs were cultured in Dulbecco’s modified Eagle’s F12 medium (DMEM/F12, Gibco), supplemented with 10% Fetal Bovine Serum (FBS, Gibco) and 1% penicillin-streptomycin (Gibco) in a humidified atmosphere containing 5% CO_2_ at 37°C. 16-HBE cells (Procell) were maintained in Dulbecco’s modified Eagle medium (DMEM, Gibco) and cultured in the same environment.

To minimize the influence of EVs, EVs-depleted FBS was employed for cell culture. FBS was initially filtered through a 0.22 µm filter membrane, followed by centrifugation in an ultra-high-speed centrifuge tube at 120,000 × g for 90 min at 4°C. The resulting supernatant, now depleted of EVs, was collected and used as EVs-depleted FBS. HucMSCs were cultured in this medium until they reached a confluency of approximately 60%-70%. At this stage, the original serum-containing medium was carefully removed, and the cells were gently washed with PBS. Subsequently, the cells were incubated in the fresh EVs-depleted FBS for an additional 48 hours. Upon achieving a confluency of 90%-95%, the supernatant was harvested for the extraction of EVs.

### Acquired and identified the differentiation capability of hucMSCs

HucMSCs were isolated from fresh umbilical cord samples using previously described methods (21). Cells in passage 3 were selected to identify surface markers associated with their mesenchymal stem cell characteristics. Cultured cells were stained with antibodies against CD11b, CD34, CD45, CD73, CD79α, CD90, CD105, and HLA-DR (BioLegend). FITC or PE-coupled mouse immunoglobulin isotype antibodies were used as negative controls. The results were detected using the FACSVerse instrument (BD Bioscience) and analyzed with FlowJo software (Tree Star). Furthermore, the adipocyte, osteoblast, and chondrogenic abilities were utilized to assess the differentiation potential of hucMSCs.

### Isolation, purification and characterization of hucMSC EVs

Cell supernatants were collected from 40 Petri dishes (diameter 100 mm) where hucMSCs were cultured. The collected samples were first centrifuged at 300 × g for 10 min to remove dead cells. The remaining supernatant was then centrifuged at 3,000 × g for 10 min to pellet apoptotic bodies and proteins. Following this, the supernatant was further centrifuged at 10,000 × g for 10 min and passed through a 0.22μm filter for purification. After ultracentrifugation at 120,000 × g for 90 min, the precipitate obtained was resuspended in PBS and subjected to the same ultracentrifugation step for further purification. The protein concentration of the EVs was used to represent the amount of “hucMSC-EVs”. The acquired pellets were then re-suspended in 1 mL sterile PBS, and the protein content was measured using a BCA Protein Assay Kit (Beyotime) following lysis with RIPA (Beyotime) in a volume ratio of 4:1. The morphology and distribution of EVs were determined using a transmission electron microscope JEM-1400 (JEOL, Japan) and nanoparticle tracking analysis (NTA) with a ZetaView^®^ PMX110 (Particle Metrix, Germany), respectively. Western blotting was performed to detect exosomal markers CD63, HSP70, and TSG101 as well as the negative marker Calnexin.

### Cell transfection

The miR-146a-5p mimics, synthesized by Ribobio, was transfected to hucMSCs using riboSCRIPTTM Reverse Transcription Kit (Ribobio). The transfection procedure was followed as manufacturer’s instructions. Then, the EVs were isolated for further detection, which named “146a-EVs”.

### EVs uptake assay

For EVs tracking, EVs were labeled using the Cell Membrane Staining Kit PKH67 (Sigma-Aldrich, USA) following the manufacturer’s instructions. Briefly, PKH67 dye was mixed with EVs and incubated in a dark environment. The mixture was then diluted with PBS and transferred to a 100 KD ultrafiltration tube (Millipore) for purification. Purified labeled EVs were observed for uptake in 16-HBE cells or mice using a confocal microscope Laika SP8.

### Allergic airway inflammation mouse model

Six-week-old female C57BL/6 mice were obtained from the Comparative Medicine Centre of Yangzhou University and housed under controlled conditions with comfortable temperature and humidity. The mice were randomly divided into three initial groups (n=6/group): (I) NC group receiving PBS as a negative control, (II) DFE group receiving *D. farinae* extract (DFE) as a positive control, and (III) DFE+hucMSC-EVs group receiving DFE in combination with hucMSC-EVs. To further investigate the role of miR-146a-5p, another group was added: (IV) DFE+146a-EVs group, which received DFE and 146a-EVs.

The DFE used in this study was purchased from Greer Laboratories (lot number: 402804). Initially, all mice, except those in the control group, were intranasally administered with 100 μg DFE resuspended in 40 μL PBS at a concentration of 2.5 μg/μL on day 0. The control group received 40 μL of PBS instead of DFE. Starting from day 7, mice were intranasally challenged with 10 μg of DFE daily for the following 5 days. The control group received PBS instead of DFE. HucMSC-EVs or 146a-EVs were resuspended in PBS and then further diluted with normal saline (NS) to achieve a concentration of 1 mg/ml. The mice were divided into different treatment groups, with the control group receiving 1 ml of NS treatment. The nebulization chamber is a rat anesthesia induction box measuring 28 cm x 21 cm x 19 cm. Both the EVs and saline inhalation treatments were given for approximately 15 minutes per day on days 6, 9, and 12 using a nebulizer (Yuwell nebulizer M102, China). On day 14, after euthanizing the mice, bronchoalveolar lavage fluid (BALF) was collected from the right lungs for cell isolation and cytokine detection via ELISA from the supernatant. The collected cells were counted. The left lung tissues were fixed with 4% paraformaldehyde, embedded in paraffin, and prepared as sections stained with H&E and PAS solution for histopathological analysis.

### Serum samples collection

Serum samples were collected from 23 children with bronchial asthma (2 to 10 years old, mean age 5.6 years) who were allergic to *D. farinae*, as well as from 15 healthy children (1 to 10 years old, mean age 4.6 years). The allergy status of the participants was confirmed by measuring the expression level of *D. farinae*-specific IgE antibodies using the CAP System (Pharmacia & Upjohn Diagnostics AB).

### RNA isolation and quantitative real-time PCR

Quantify the levels of miR-146a-5p in EVs and miRNAs in serum: total RNA was extracted from miR-146a-EVs and human serum samples using the EasyPure^®^ RNA Kit (TransGen Biotech, Beijing, China). The quantity of extracted RNA was assessed using a NanoDrop Ultramicro spectrophotometer (Thermo Fisher Scientific). Subsequently, miRNAs were reverse transcribed into cDNA with the riboSCRIPT Reverse Transcription Kit (RiboBio, Guangzhou, China). The expression levels of miRNAs were then quantified using the miDETECT A Track™ miRNA qRT-PCRStarter Kit (RiboBio) with primers also designed by RiboBio, on the Applied Biosystems^®^ 7500 Real-time PCR Systems (Thermo Fisher Scientific). The relative expression of miRNAs was normalized to *cel-39* (RiboBio) and calculated using the 2^-ΔΔct^ method.

Quantify the levels of mRNA in 16-HBE cells and lung tissues: total RNA was extracted from 16-HBE cells and lung tissue using RNAiso Plus (Takara) following the manufacturer’s protocol. The extracted RNA was then transcribed into cDNA using a cDNA synthesis kit (Takara). Quantitative real-time PCR (qRT-PCR) was performed using SYBR Green (Takara), and the amplification results were calculated using the 2^−ΔΔCt^ method. The primer sequences specific to the target genes are listed in **Table 1**. All experiments were repeated at least twice.

**Table 1:** Primer sequences.

Targets	Primer sequence	
	Forward	Reverse
hum-TRAF6	5’- ATGCGGCCATAGGTTCTGC-3’	5’- TCCTCAAGATGTCTCAGTTCCA-3’
hum-IRAK1	5’- GTCAGAGCCACCGCAGATTA-3’	5’- CCTCCTCACTGGATGATGCC-3’
mus-TRAF6	5’- CATCTTCAGTTACCGACAGCTCAG-3’	5’- TGGTCGAGAATTGTAAGGCGTAT-3’
mus-IRAK1	5’- GCTGTGAAGAGACTGAAGGAGGA-3’	5’- ACGATATTTGGGTGACGAAACC-3’
β-actin	5’- GGCTGTATTCCCCTCCATCG-3’	5’- CCAGTTGGTAACAATGCCATGT-3’

### Western blotting

Protein was extracted using RIPA lysis buffer, and the protein concentration was quantified using a BCA Protein Assay Kit (Beyotime). Equal amounts of protein were then separated on an SDS-PAGE gel and transferred to polyvinylidene fluoride (PVDF) membranes. After blocking with 5% skim milk for 1 hour, the primary antibody was applied overnight at 4°C, followed by application of the secondary antibody for 1 hour at room temperature. The protein expression levels were detected by applying an ECL substrate (Thermo). Primary antibodies were all purchased from Abcam and listed as follows: TSG101 (ab133586), CD63 (ab134045), HSP70 (ab2787), Calnexin (ab22595), NLRP3 (ab263899), NF-κB (ab32360), IRAK1 (ab238), TRAF6 ab33915), and β-actin (ab8227). The procedure for calculating the grayscale values of Western blot bands using Image J, a software developed by the National Institutes of Health.

### Enzyme-linked immunosorbent assay

Cytokines were measured using commercial ELISA kits following the manufacturer’s instructions. The cytokines that were detected included: IL-1β (4A Biotech, IB99537), IL-4 (4A Biotech, IB09619), IL-5 (4A Biotech, IB99547), IL-6 (4A Biotech, IB99549; PharmaGenie, HUDC0062-96), IL-10 (4A Biotech, CME0016-048), IL-18 (Biosensis, BEK-2119-1P), IL-33 (PharmaGenie, HUDC0056-96), TNF-α (AssayGenie, MOES00668; PharmaGenie, HUDC0073-96), and IgE (EMIGHEX5, Invitrogen).

### Dual-luciferase assay

The 3’-UTR of IRAK1 and TRAF6 containing the predicted miR-146-5p seed-matching site was amplified from a cDNA library using PCR. The amplified sequences were then cloned into pmiR-REPORT-3’-UTR vectors, which contained both wild type (WT) and mutant type (Mut) inserts. Simultaneously, miR-146a-5p mimics and negative control miRNA (Ribobio) were transfected into 16-HBE cells. After 48 hours of transfection, the 16-HBE cells were lysed using a dual luciferase assay kit (Promega), and luciferase activities were measured and normalized to the control. The fold change in luciferase activity for each miRNA compared to the negative control was calculated.

### Immunofluorescence

To track the nuclear translocation of NF-κB p65, 16-HBE cells were co-cultured with hucMSC-EVs or 146a-EVs. After co-culturing, the cells were fixed, permeabilized, blocked, and then incubated overnight at 4°C with the primary antibody against NF-κB p65 (Abcam, ab16502). Subsequently, the cells were rinsed with PBS and incubated with the secondary antibody, Anti-rabbit IgG (H+L), F(ab’)2 Fragment (Cell Signaling Technology, #4412), at room temperature for 1 hour. After a final rinse, the nuclei were stained with 4’,6-diamidino-2-phenylindole (DAPI) purchased from Thermo) for 30 minutes. All images were captured using a Laika SP8 confocal microscope and analyzed using Image J software.

### Statistical analysis

The data are presented as the mean value ± standard deviation (SD). An unpaired two-tailed Student’s t-test was performed for comparisons between two groups, while one-way analysis of variance (ANOVA) with the Tukey test was performed for multiple comparisons. A p-value of less than 0.05 (*P* < 0.05) was considered statistically significant. All statistical analyses were performed using GraphPad software version 9.0 (Prism, La Jolla, CA, USA).

## Results

### Isolation and characterization of hucMSCs and hucMSC-EVs

**Figure 1:**
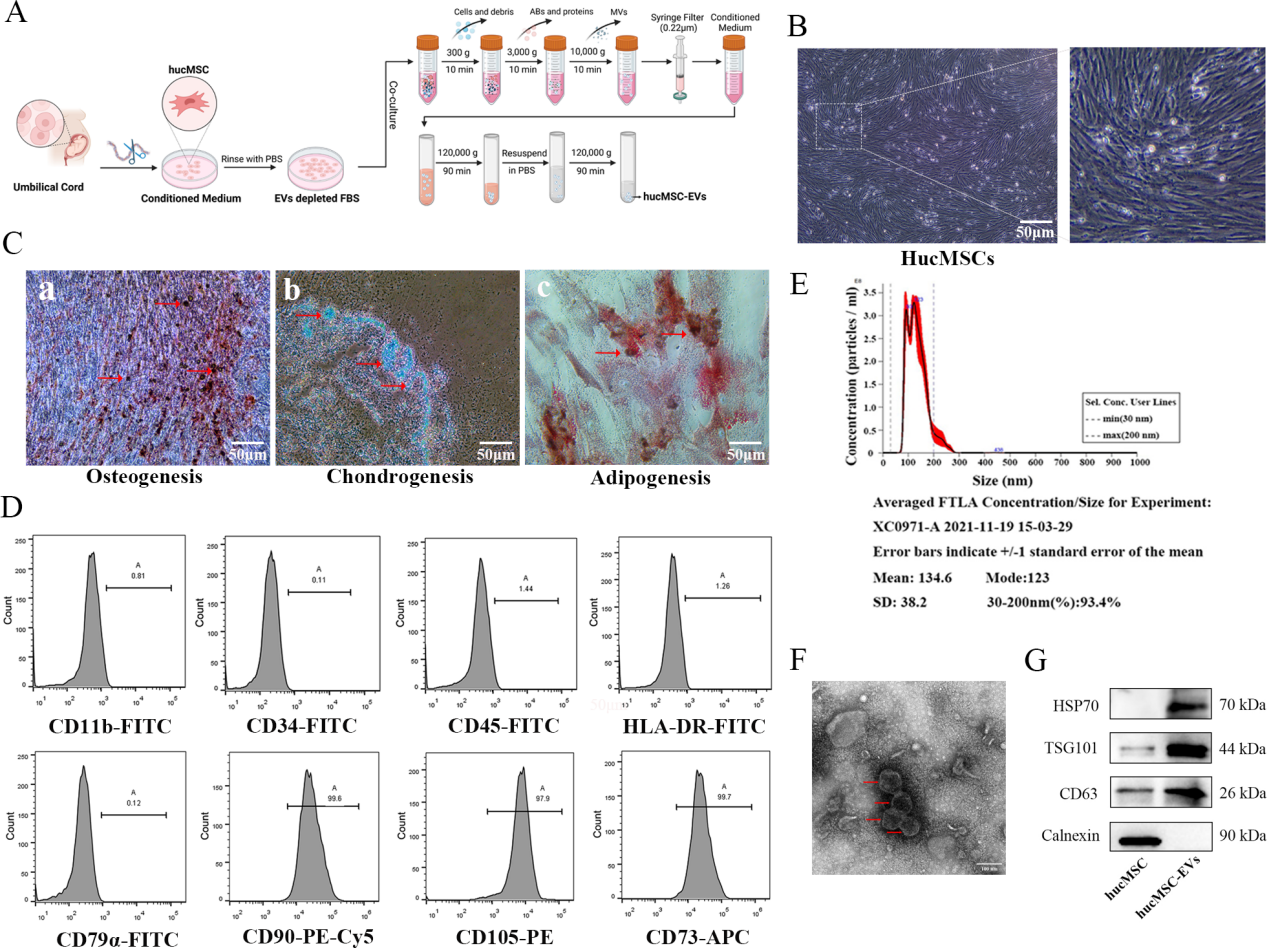
Identification of hucMSCs and derived EVs. **(A)** The flowchart for acquisition and culture of hucMSCs, as well as the attainment of hucMSCs derived extracellular vesicles (hucMSC-EVs) via differential ultracentrifugation approach. **(B)** The cell morphology of hucMSCs (passage 3) was observed under a light microscope (scale bar, 50μm). **(C)** Representative images of osteocytes (a), chondrocytes (b) and adipocytes (c) differentiation of hucMSCs cultured in the differentiation media. The cells were analyzed using cytochemical staining with Alizarin Red, Arsenic Blue and Oil Red O (scale bar, 50 μm). **(D)** Flow cytometry analysis of hucMSC surface markers. **(E)** The distribution of the size for hucMSC-EVs was examined using a ZetaView. **(F)** Morphological photos of typical hucMSC-EVs under transmission electron microscope, (scale bar, 100 nm). **(G)** Western blotting analysis for positive hucMSC-derived exosomal markers (HSP70, TSG101 and CD63) and negative exosomal marker (Calnexin).

The process of hucMSCs culture and EVs extraction, as described in **Figure 1A**, can be summarized as follows: hucMSCs are obtained using adherent culture method, and EVs are extracted using differential and ultracentrifugation. The inverted phase-contrast microscope image (**Figure 1B**) displayed the typical characteristics of stem cells, including fibrous and swirling growth patterns. To confirm their identity, induced differentiation experiments were conducted and demonstrated that the hucMSCs could differentiate into osteoblasts, chondrocytes, and adipocytes (**Figure 1C**). Flow cytometric analysis showed positive expression of CD73, CD90, and CD105, while CD11b, CD34, CD45, CD79, and HLA-DR exhibited very low expression in the cultured cells, consistent with the expression profile of stem cells (**Figure 1D**). After differential and ultracentrifugation to extract extracellular vesicles from the conditioned medium of hucMSCs, the particle size distribution of nanoparticles was determined using NTA, which resulted in a mean size of 134.6 ± 38.2 nm (**Figure 1E**). Transmission electron microscopy images (**Figure 1F**) displayed round-cup-shaped nanoparticles with diameters of approximately 100 nm. Western blotting confirmed the expression of exosomal surface marker proteins CD63, TSG101, and HSP70, while Calnexin showed negative expression (**Figure 1G**). Consequently, these nanoparticles were identified as hucMSC-EVs.

### HucMSC-EVs attenuated DFE-induced allergic airway inflammation in mice

**Figure 2:**
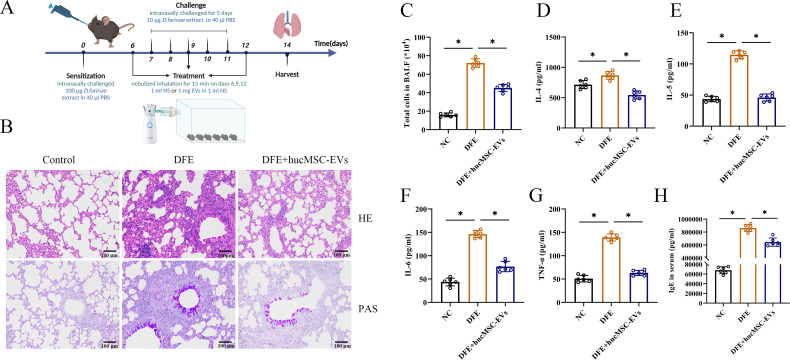
HucMSC-EVs attenuated *D*. *farinae*-induced allergic airway inflammation in mice. **(A)** Schematic diagram for establishing mouse models of *D*. *farinae* extract sensitized allergic airway epithelial cell inflammation; n=6 biological independent animals per group. **(B)** Pathological changes in mice lung tissues measured by H&E and PAS staining (scale bar, 100 μm). **(C)** Total number of cells in BALF (n=6). **(D–H)** Expression levels of IL-4, IL-5, IL-6 and TNF-α in the BALF and serum levels of allergen-specific IgE, measured by ELISA (n=6). *P<0.05. ANOVA with the Tukey test was performed for comparison between three groups.

To investigate the potential therapeutic effect of hucMSC-EVs on DFE induced allergic airway inflammation, a mouse model of allergic airway inflammation was established (**Figure 2A**). Histopathological examination of the lungs showed obvious perivascular and interstitial infiltration of inflammatory cells in the DFE group, which was attenuated by treatment with hucMSC-EVs (**Figure 2B**). In BALF, the total number of inflammatory cells was increased in the DFE group compared to the NC group, but this increase was alleviated by treatment with hucMSC-EVs (**Figure 2C**). Moreover, the concentrations of IL-4, IL-5, IL-6, and TNF-α in BALF were elevated in the DFE group, but showed a comparative decrease in the DFE+hucMSC-EVs group (**Figures 2D–G**). Similarly, the expression levels of IgE in the mice serum exhibited a similar pattern, demonstrating that hucMSC-EVs was able to reverse the elevated IgE caused by DFE (**Figure 2H**). These findings provide evidence that hucMSC-EVs have the ability to attenuate *D. farinae*-induced allergic airway inflammation.

### Attenuation of inflammatory cytokines release in 16-HBE cells induced by DFE through administration of hucMSC-EVs

**Figure 3:**
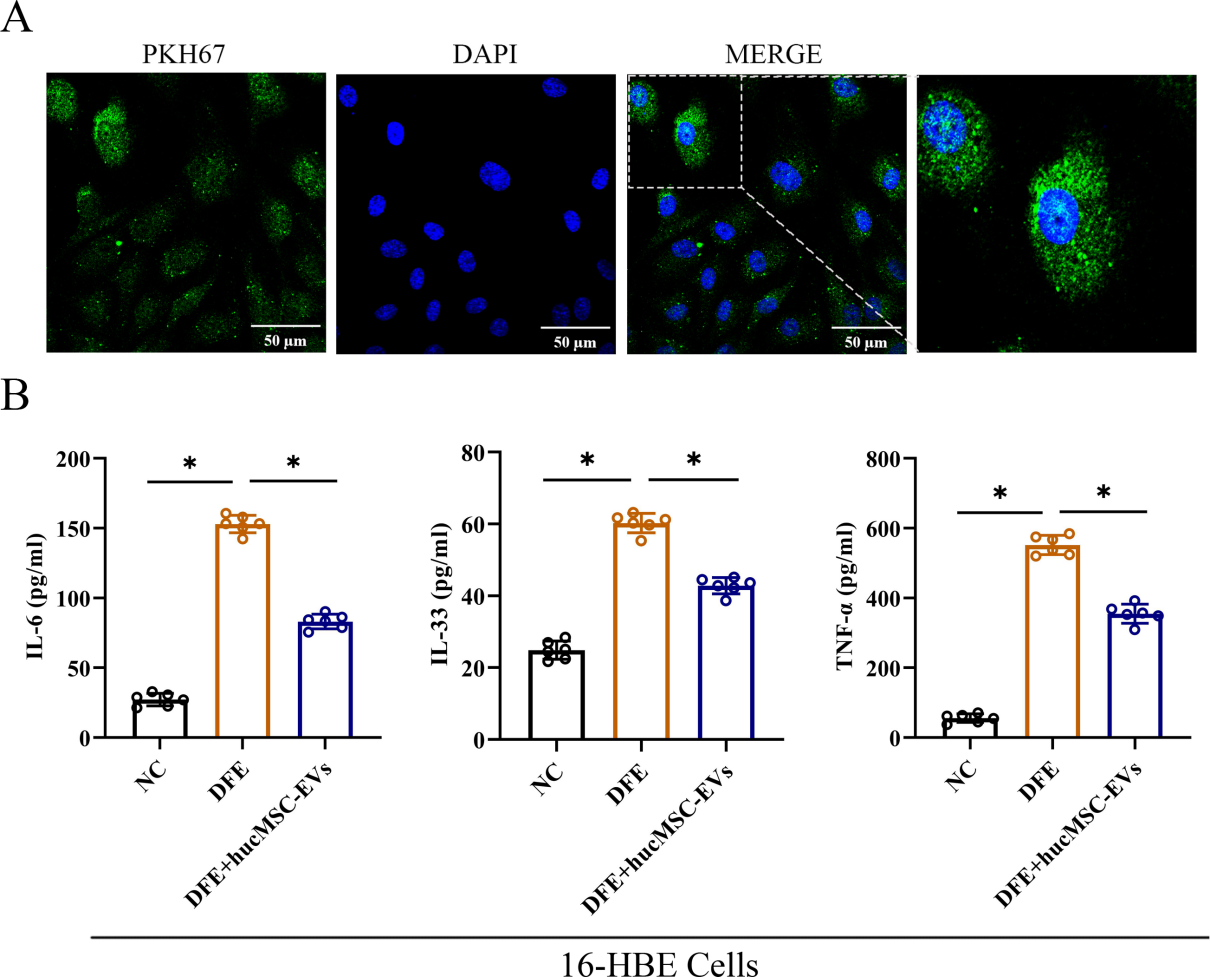
HucMSC-EVs attenuated *D*. *farinae*-induced allergic inflammation in 16-HBE cells. **(A)** Uptake of hucMSC-EVs was confirmed in 16-HBE cells by fluorescence microscopy (scale bar, 50 μm). **(B)** Levels of IL-6, IL-33 and TNF-α in the cell supernatants were detected by ELISA (n=6). *P<0.05. ANOVA with the Tukey test was performed for comparison between three groups.

To investigate the effects of hucMSC-EVs *in vitro*, 16-HBE cells were utilized to further confirm the findings. The results demonstrated that PKH67-labeled hucMSC-EVs could be internalized by 16-HBE cells (**Figure 3A**). Additionally, as depicted in **Figure 3B**, the expression levels of IL-6, IL-33, and TNF-α were markedly elevated in the DFE group. However, upon administration of hucMSC-EVs, the expression levels of these inflammatory cytokines were reduced. Hence, these findings suggest that hucMSC-EVs possesses the ability to alleviate DFE induced the release of inflammatory cytokines in 16-HBE cells.

### Correlation between decreased miR-146a-5p expression and DFE stimulation

**Figure 4:**
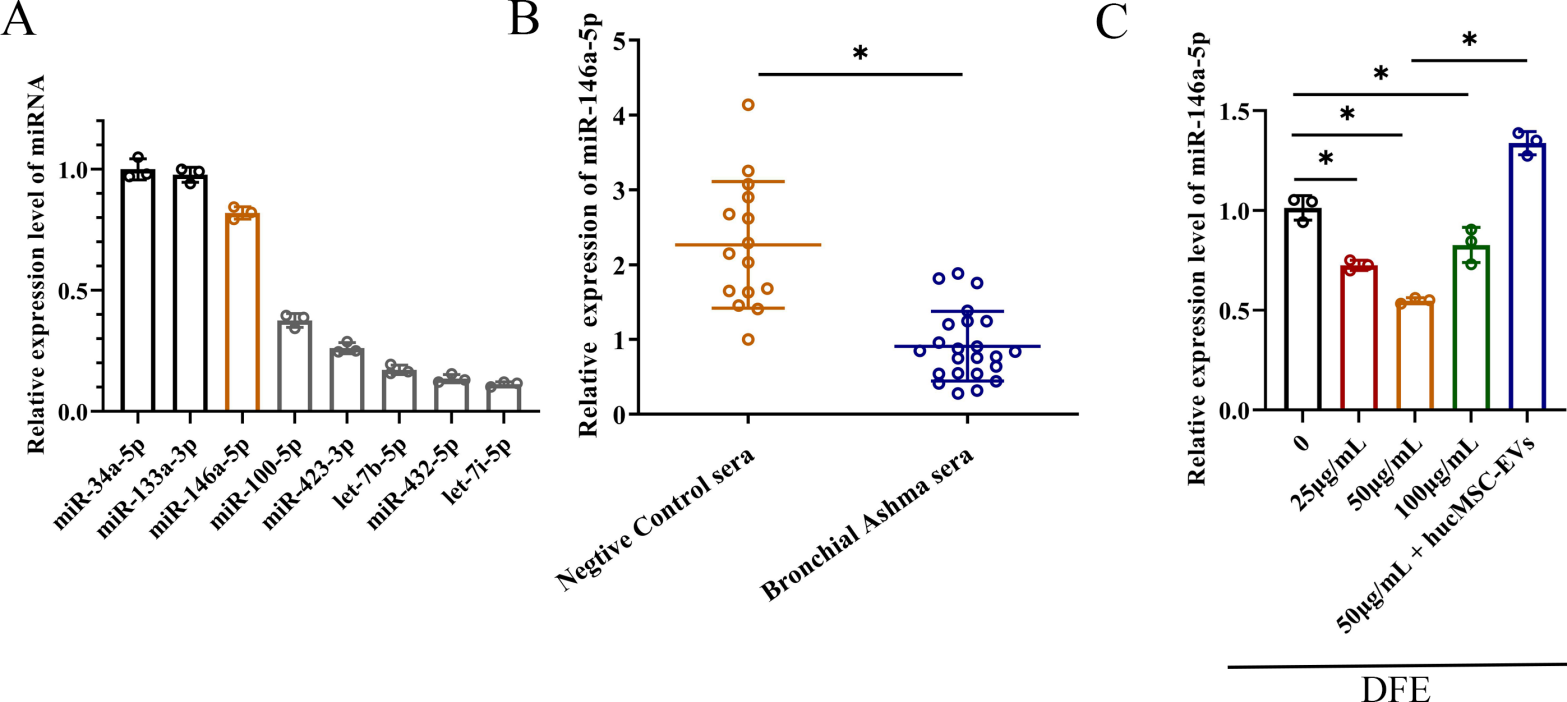
The decrease of miR-146a-5p was relevant with *D*. *farinae* extract stimulation. **(A)** Relative expression levels of miRNAs that were highly expressed in hucMSCs (n=3). **(B)** Relative expression levels of miR-146a-5p in serum from healthy children (n=15) and bronchial asthma children (n=23) allergic to *D*. *farinae*. **(C)** Relative expression levels of miR-146a-5p in 16-HBE cells following stimulation with varying concentrations of *D*. *farinae* extracts (DFE) and administration of hucMSC-EVs (n=3). *P<0.05. An unpaired two-tailed Student’s t-test was performed for comparisons between healthy and bronchial asthma children, while ANOVA with the Tukey test was performed for comparison between five groups.

Numerous studies have shown that MSC derived EVs play a crucial role in mediating anti-inflammatory effects through cell-to-cell interactions. In order to achieve better anti-inflammatory effect, combined with our previous experimental data as well as the sequencing data from hucMSCs (20), we selected miRNAs that were highly expressed in hucMSCs, and carried out the relevant quantitative validation assay (**Figure 4A**). The miRNAs that obviously enriched in hucMSC-EVs included miR-34a-5p, miR-133a-3p, miR-146a-5p, miR-100-5p, miR-423-3p, let-7b-5p and miR-432-5p. Therefore, due to relatively high expression levels and anti-inflammatory properties, miR-146a-5p was selected as the target gene for upregulation in engineered hucMSC-derived EVs, aiming to achieve an enhanced anti-inflammatory effect.

To preliminarily investigate the association between miR-146a-5p and allergic airway inflammation, we collected serum samples from healthy children as the negative control group and children with bronchial asthma who were specifically allergic to *D. farinae*. As shown in **Figure 4B**, we observed a lower relative expression of miR-146a-5p in the serum of children allergic to *D. farinae* compared to healthy children.

Based on the previous results of cell and mouse experiments, we intended to further explore miR-146a-5p expression level in epithelial cells sensitized with different concentrations of DFE (**Figure 4C**). Results showed that the expression level of miR-146a-5p was reduced by the stimulation of DFE, and expressed lowest level at the concentration of 50 μg/ml. Nevertheless, the application of hucMSC-EVs was able to reverse the expression of miR-146a-5p.

Collectively, we can conclude that decreased miR-146a-5p expression correlates with DFE stimulation.

### MiR-146a-5p-engineered hucMSC-derived EVs can more significantly reduce the release of inflammatory cytokines in 16-HBE cells

**Figure 5:**
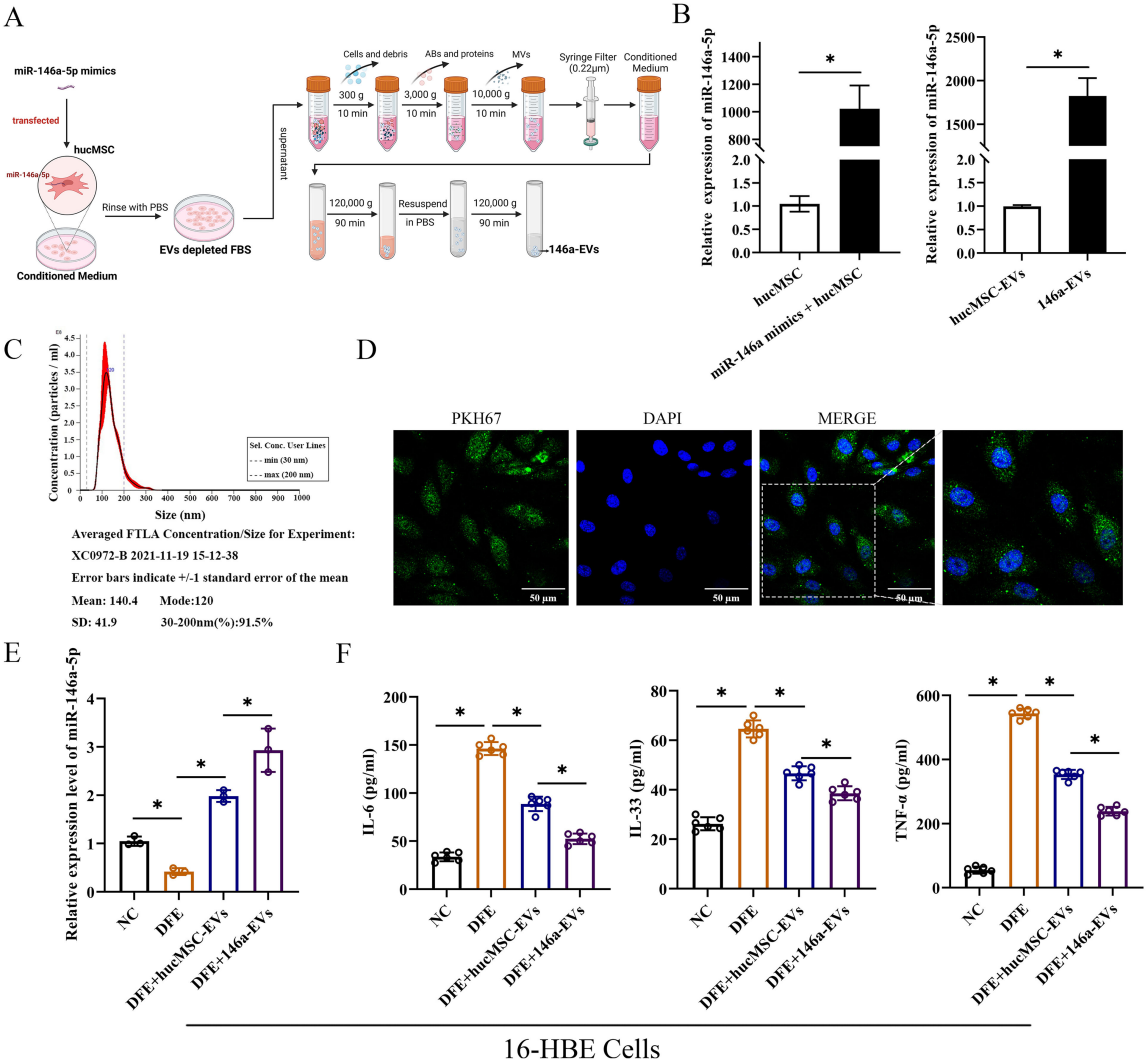
MiR-146a-5p-engineered hucMSC-derived EVs can more significantly reduce the release of inflammatory cytokines in 16-HBE cells. **(A)** Schematic illustration of the modification and isolation of miR-146a-5p-engineered hucMSC-derived EVs (146a-EVs). **(B)** Relative expression levels of miR-146a-5p in hucMSCs and EVs after miR-146a-5p mimics transfection. **(C)** Size distribution of the 146a-EVs. **(D)** Uptake of 146a-EVs tagged with PKH67 (green) in 16-HBE cells determined by fluorescence microscopy (scale bar, 50 μm). **(E)** Relative expression levels of miR-146a-5p in DFE-sensitized 16-HBE cells after treatment of hucMSC-EVs or 146a-EVs (n=3). **(F)** Levels of IL-6, IL-33 and TNF-α in the cell supernatant, detected by ELISA. (n=6). *P<0.05. An unpaired two-tailed Student’s t-test was performed for comparisons between two groups. ANOVA with the Tukey test was performed for comparison between four groups.

To enhance the therapeutic effects of hucMSC-EVs, hucMSCs were transfected with miR-146a-5p mimics in conditioned medium. The supernatant was then centrifuged, filtered, and purified to obtain EVs with overexpressed miR-146a-5p (referred to as 146a-EVs) (**Figure 5A**). First, the expression of miR-146a-5p was measured to verify the efficiency of the constructed 146a-EVs (**Figure 5B**). In hucMSCs, the group that received miR-146a-5p mimics showed significant overexpression of miR-146a-5p, and the expression of miR-146a-5p was also higher in the 146a-EVs group compared to the hucMSC-EVs group. Next, NTA demonstrated that the mean size of 146a-EVs was similar to hucMSC-EVs, measuring 140.4 ± 41.9 nm (**Figure 5C**). Additionally, the ability of 146a-EVs to be internalized into 16-HBE cells was confirmed (**Figure 5D**).

To investigate whether miR-146a-5p-engineered hucMSC-derived EVs could more significantly attenuate *D. farinae*-induced airway inflammation *in vitro*, we first confirmed that there was a high expression of miR-146a-5p in hucMSC-EVs. Notably, compared to hucMSC-EVs treatment, 146a-EVs showed a more pronounced increase in miR-146a-5p expression in 16-HBE cells treated with DFE (**Figure 5E**). As shown in **Figure 5F**, the levels of inflammatory cytokines IL-6, IL-33, and TNF-α were more significantly reduced in the DFE+146a-EVs group compared to the DFE+hucMSC-EVs group. Therefore, these findings indicate that overexpressed miR-146a-5p can enhance the ability of hucMSC-EVs to attenuate *D. farinae*-induced inflammation in airway epithelial cells. We will further investigate the underlying mechanisms behind this enhancement in subsequent experiments.

### MiR-146a-5p-engineered hucMSC-derived EVs inhibit the NF-κB/NLRP3 pathway through regulation of IRAK1/TRAF6 and effectively alleviate *D. farinae*-induced inflammation *in vitro*

**Figure 6:**
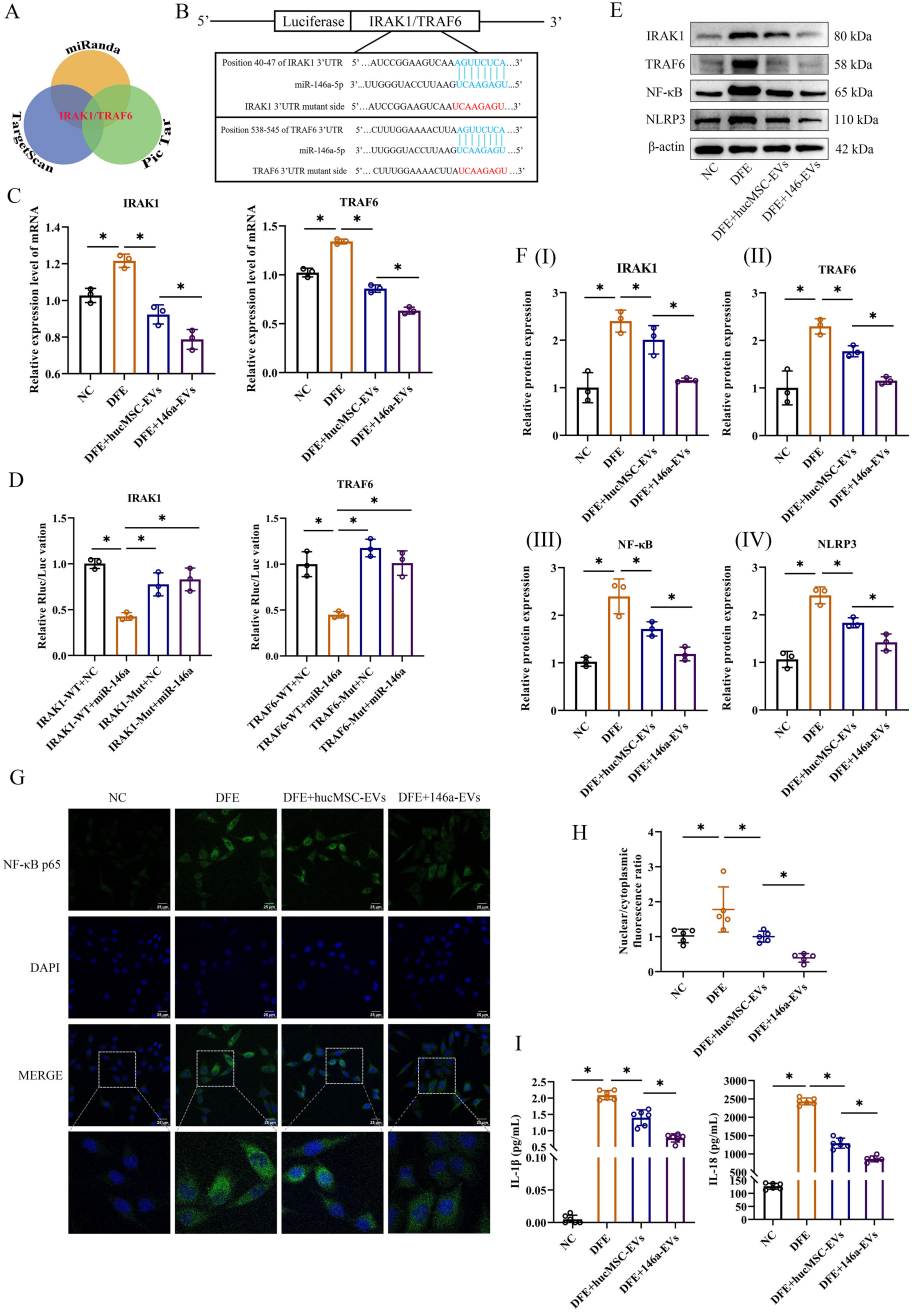
146a-EVs inhibit the NF-κB/NLRP3 pathway through regulation of IRAK1/TRAF6 and effectively alleviate *D*. *farinae*-induced inflammation *in vitro.* **(A)** Targeting mRNAs of miR-146a-5p predicted on Pic Tar, TargetScan and miRanda. **(B)** Predicted miR-146a-5p binding site (blue) in wild-type IRAK1 or TRAF6 3’UTR and mutant sequence (red). **(C)** Relative mRNA expression levels of IRAK1 or TRAF6 in 16-HBE cells treated with hucMSC-EVs or 146a-EVs (n=3). **(D)** Results of luciferase reporter assay by co-transfecting a luciferase vector containing the wild-type (WT) or mutant (MUT) 3’UTR of IRAK1 or TRAF6 together with miR-146a-5p mimics or negative control miRNA (n=3). **(E, F)** Relative protein expression levels of IRAK1, TRAF6, NF-κB and NLRP3 in 16-HBE cells treated with hucMSC-EVs or 146a-EVs (n=3). **(G, H)** Nuclear translocation of NF-κB p65 (green) in 16-HBE cells treated with hucMSC-EVs or 146a-EVs was confirmed by fluorescence microscopy (n=5, scale bar = 25 μm). **(I)** Levels of IL-1β and IL-18 in the supernatant of cultured 16-HBE cells treated with hucMSC-EVs or 146a-EVs, detected by ELISA (n=6). Data were expressed as mean ± SD. *P<0.05. ANOVA with the Tukey test was performed for comparison between four groups.

To further elucidate better therapeutic effect of miR-146a-5p-engineered hucMSC-derived EVs in alleviating airway inflammation, an extensive search was conducted using three public miRNA databases (Pic Tar, TargetScan, and miRanda) to identify potential target genes of miR-146a-5p that may contribute to attenuate inflammation (**Figure 6A**). The findings revealed that IRAK1 and TRAF6 could serve as potential targets for miR-146a-5p regulation. In addition, the probable binding sites between miR-146a-5p and the 3’ UTR regions of IRAK1/TRAF6 mRNA were predicted (**Figure 6B**). Following successful transfection, 16-HBE cells were stimulated with DFE, and qRT-PCR results demonstrated significant suppression of IRAK1 and TRAF6 mRNA expression levels upon treatment with hucMSC-EVs or 146a-EVs. Notably, the DFE+146a-EVs group exhibited a more pronounced decrease in IRAK1 and TRAF6 mRNA expression levels (**Figure 6C**).

To assess the direct regulatory effect, a luciferase reporter assay was performed by co-transfecting luciferase vectors containing wild-type (WT) or mutant (MUT) 3’UTR regions of IRAK1 or TRAF6, along with miR-146a-5p mimics or negative control miRNA. Overexpression of miR-146a-5p resulted in a significant reduction in luciferase activity in the WT group of 16-HBE cells. However, luciferase activity was completely restored when mutant IRAK1 or TRAF6 3’UTR regions were introduced (**Figure 6D**). These findings suggest that miR-146a-5p can directly regulate the expression of IRAK1 and TRAF6 through their 3’UTR regions.

In the study of inflammatory disease mechanisms, IRAK1 or TRAF6 has been demonstrated to regulate downstream NF-κB p65 subunit nuclear translocation, which is a classical inflammatory pathway (22). NF-κB not only promotes the expression of NLRP3 inflammasome, but also upregulates the expression of inflammatory cytokines, such as IL-2, IL-6, IL-8, IL-18, IL-1β and TNF-α (23). Based on these researches, we measured relative protein expressions of IRAK1, TRAF6, NF-κB and NLRP3 *in vitro* by western blotting, as well as inflammatory cytokines in our study. In **Figures 6E, F**, the results of western blotting revealed a notable reduction in inflammation following treatment with hucMSC-EVs or 146a-EVs in 16-HBE cells exposed to DFE. Furthermore, the anti-inflammatory effect was more pronounced in the 146a-EVs group. Also, IF analysis of 16-HBE cells showed DFE stimulation significantly promoted NF-κB nuclear translocation, while hucMSC-EVs could downregulate this translocation, and 146a-EVs had more obvious downregulation effect (**Figures 6G, H**). Similarly, the results showed the same trend after detection of inflammatory cytokines IL-1β and IL-18 (**Figure 6I**). Collectively, these findings suggested that miR-146a-5p-engineered hucMSC-derived EVs suppressed inflammation *in vitro* more effectively by inhibiting the expression of IRAK1 and TRAF6 and then regulating NF-κB/NLPR3 signal pathway.

### miR-146a-5p engineered hucMSC-derived EVs exert more potent anti-inflammatory effects *in vivo* by regulating NF-κB/NLRP3 via the IRAK1/TRAF6 pathway

**Figure 7:**
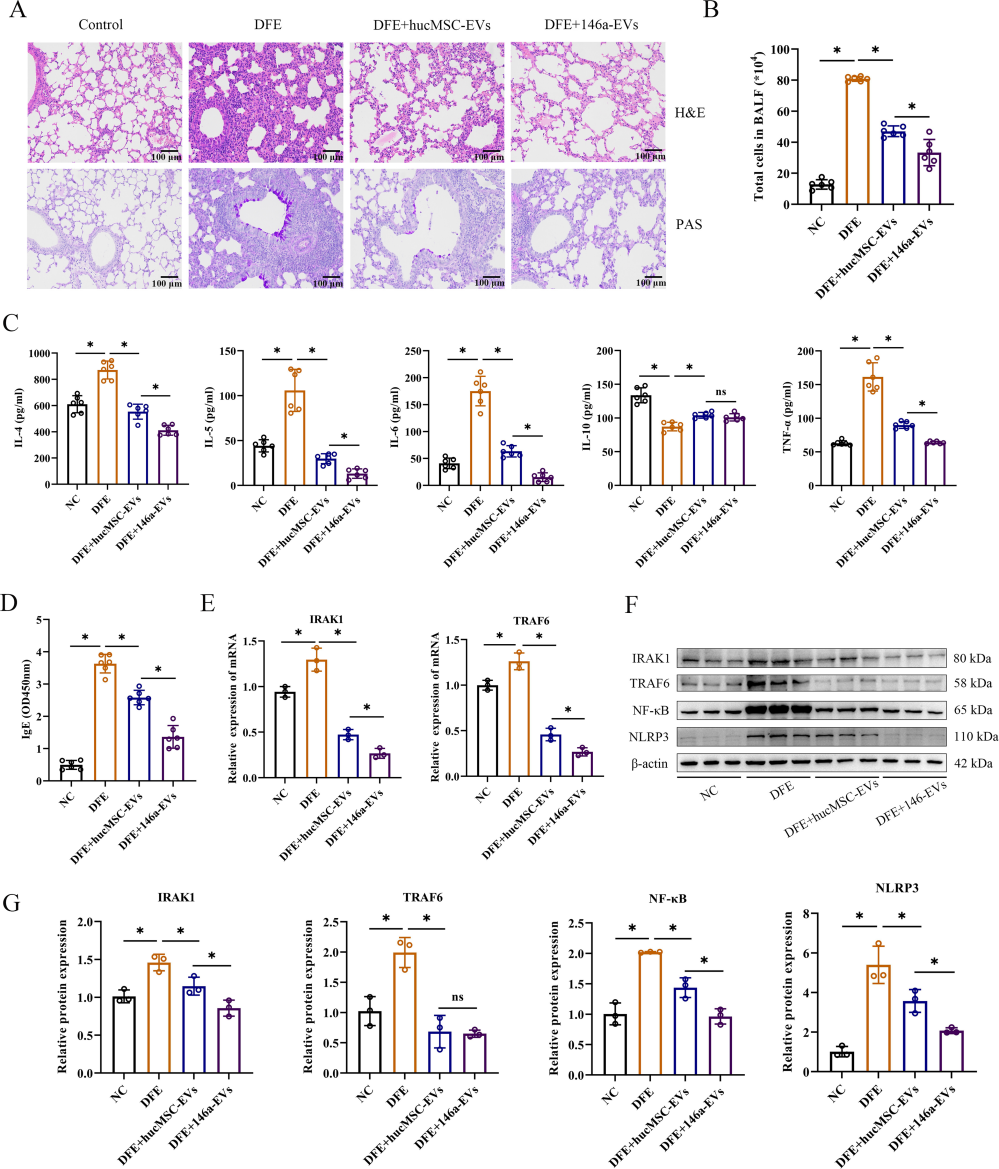
miR-146a-5p engineered hucMSC-derived EVs exert more potent anti-inflammatory effects *in vivo* by regulating NF-κB/NLRP3 via the IRAK1/TRAF6 pathway. **(A)** Pathological changes in mouse lung tissues measured by H&E and PAS staining (scale bar = 100 μm). **(B)** Total number of cells in BALF (n=6). **(C, D)** Levels of IL-4, IL-5, IL-6, IL-10, TNF-α in BALF, and IgE in the mice serum, detected by ELISA (n=6). **(E)** Relative mRNA expression levels of IRAK1 or TRAF6 in lung tissues treated with hucMSC-EVs or 146a-EVs (n=3). **(F, G)** Relative protein expression levels of IRAK1, TRAF6, NF-κB and NLRP3 in mice detected by Western blotting (n=3). Data were expressed as mean ± SD. ns, not significant. *P<0.05. ANOVA with the Tukey test was performed for comparison between four groups.

To investigate if the overexpression of miR-146a-5p enhances the inhibitory effect of hucMSC-EVs on allergic inflammation *in vivo*, we administered hucMSC-EVs or 146a-EVs to mice treated with DFE and examined their anti-inflammatory effects. As expected, mice treated with aerosolized 146a-EVs exhibited more pronounced beneficial effects on airway inflammation compared to those treated with hucMSC-EVs. In lung tissues, the DFE+146a-EVs group showed fewer infiltrating inflammatory cells compared to the DFE+hucMSC-EVs group (**Figure 7A**). Additionally, in **Figures 7B–D**, it can be observed that the total cells count in BALF, expression levels of pro-inflammatory cytokines (IL-4, IL-5, IL-6, and TNF-α) in BALF, and serum IgE levels declined more significantly in the DFE+146a-EVs group compared to the DFE+hucMSC-EVs group. The anti-inflammatory cytokine IL-10 exhibited reduced expression in the BALF of mice treated with DFE. However, treatment with hucMSC-EVs and 146a-EVs was able to elevate the levels of IL-10 that had been decreased by DFE, with no significant difference in effect between the two treatments. We assessed the mRNA expression of IRAK1 and TRAF6 by qRT-PCR (**Figure 7E**) and demonstrated that DFE upregulated the expression of both genes compared to the non-challenged group, whereas treatment with hucMSC-EVs or 146a-EVs downregulated their expression, with a more significant reduction observed in the 146a-EVs group. Moreover, western blotting revealed that the protein expression levels of IRAK1, TRAF6, NF-κB, and NLRP3 decreased in the lungs of *D. farinae*-challenged mice after treatment with hucMSC-EVs and 146a-EVs (**Figures 7F, G**), with a more pronounced effect observed in the latter group.

**Figure 8:**
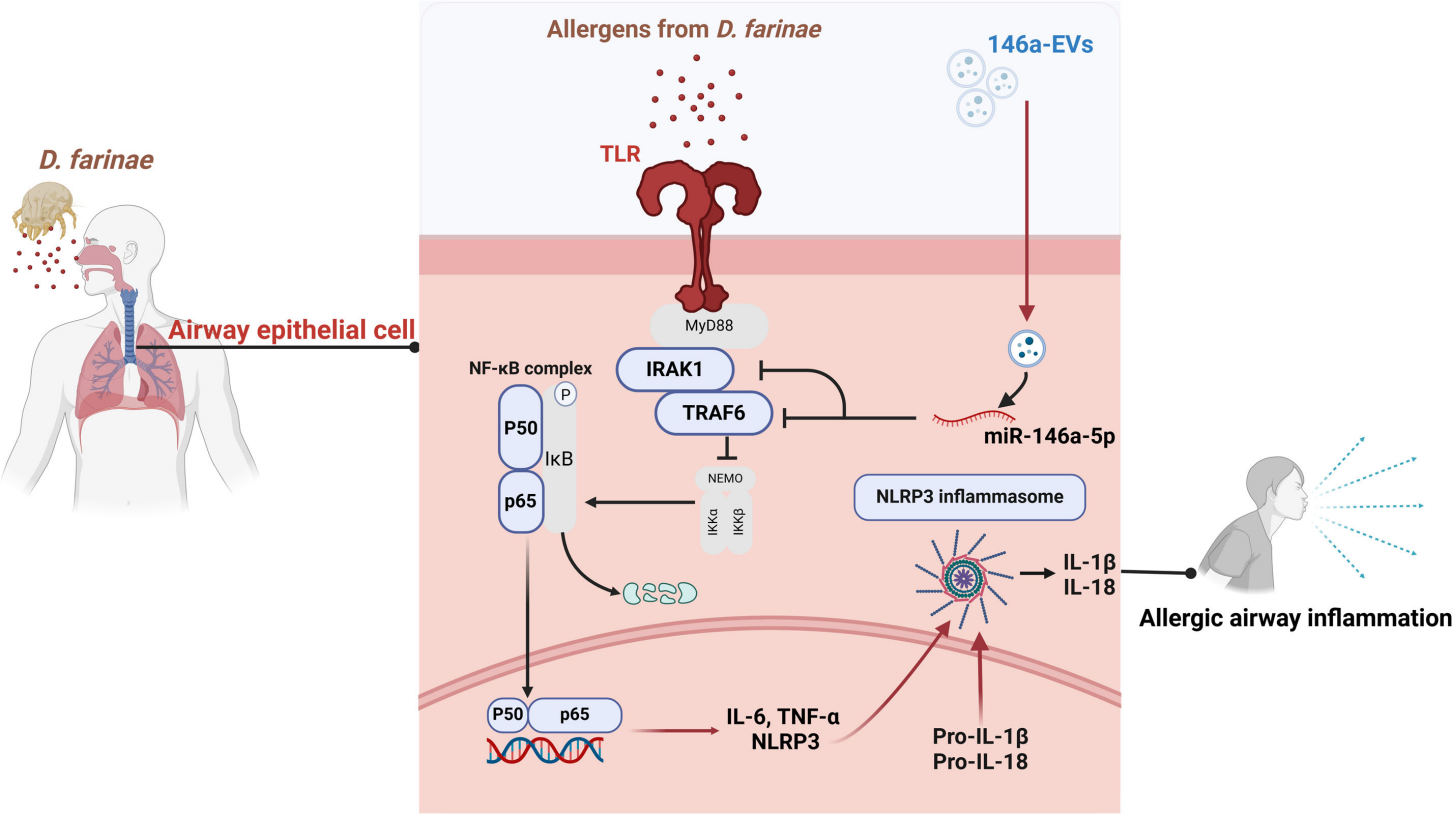
Schematic illustration of miR-146a-5p engineered hucMSC-derived EVs attenuated the *D. farinae*-induced allergic airway epithelial cell inflammation.

The result of *in vivo* experiment showed that aerosolized 146a-EVs had better anti-inflammatory effects in the mouse model of *D. farinae*-induced allergic airway inflammation compared to hucMSC-EVs. In summary, the mouse model further confirmed the better anti-inflammatory effects of miR-146a-5p-engineered hucMSC-derived EVs for alleviating *D. farinae*-induced allergic airway inflammation (**Figure 8**).

## Discussion

Allergic asthma is a prevalent inflammatory disease with significant impact on global health (24, 25). Current clinical approaches only provide symptom relief and cannot cure the disease. In our study, we aimed to overcome these limitations by developing miRNA-engineered EVs derived from hucMSCs, which demonstrated superior anti-inflammatory effects in the treatment of allergic asthma induced by *D. farinae*. **Figure 8** illustrates our research workflow and experimental procedures.

EVs, compared to traditional nanocarriers, offer a natural and efficient approach for safe delivery in cell-free therapies for various diseases (26). Engineered EVs have been successfully employed in treating diverse conditions. For instance, they can deliver anti-tumor drugs to specific cancer sites, resulting in reduced treatment-related adverse effects (27). Additionally, engineered exosomes delivering miR-140 and acting on chondrocytes, which is promising to be a novel treatment for osteoarthritis (28). HucMSC-EVs, derived from human umbilical cord mesenchymal stem cells, have emerged as a frequently studied option in regenerative medicine and the treatment of various diseases due to their non-invasive collection method, high proliferation rate, and low immunogenicity (26). Notably, hucMSC-EVs have demonstrated significant potential in mitigating inflammatory responses in patients with traumatic brain injuries (29). Engineered hucMSC-EVs, as a safe therapeutic mediator and the main focus of our study, were delivered to the airways via nebulization, revealing enhanced anti-inflammatory effects in bronchial epithelial cells. As reported, the administration of EVs through inhalation nebulization enables more efficient delivery of mRNA and protein cargoes to the bronchioles and alveoli, along with improved retention in lung tissue, which enhances the bioavailability of mRNA and protein therapeutics within the lung (30). Considering the accessibility of huc-MSC-EVs, the benefits of this delivery method, and their outstanding anti-inflammatory characteristics, miR-146a-engineered hucMSC-EVs shows potential for clinical use in the management of allergic asthma.

Emerging experimental evidence suggests that miR-146 plays a crucial role in regulating allergic inflammatory diseases (31). Studies have demonstrated that miR-146a can alleviate chronic skin inflammation in atopic dermatitis by suppressing innate immune responses (32). Furthermore, miR-146a has shown therapeutic potential in rheumatoid arthritis, and researchers have verified its ability to enhance the immunomodulatory effects of MSC-derived EVs in this condition (33). Additionally, investigations have revealed that overexpressing miR-146a has a potent anti-inflammatory effect in a mouse model of allergic airway inflammation (18). Based on these findings, we engineered hucMSC-derived EVs with miR-146a-5p to enhance their anti-allergic and anti-inflammatory efficacy.

In previous studies, researchers investigated allergic airway inflammation with a dominance of ILC2 by using different stimuli such as OVA solution or murine IL-33 to construct mouse models (20, 34). However, it is worth noting that epidemiological studies have identified indoor allergens like HDMs and pollen as the highest risk factors for the onset of asthma (35, 36). In recent years, the airway epithelium has been increasingly recognized as a critical component in the initiation of immune-inflammatory responses in asthma. As an immunologically active barrier, the airway epithelium expresses pattern recognition receptors that detect environmental stimuli and trigger inflammatory responses (37, 38). To mimic human sensitization in daily life as closely as possible, we chose DFE to sensitize mice through intranasally instillation. Additionally, we employed nebulized delivery of EVs to simulate the nebulized treatment commonly used in patients with allergic asthma. However, the limitation should be noted, we could not assess the amount of nebulized EVs in mice accurately. To mimic the nebulization treatment method used in real-life for allergic asthma patients, the medication was administered at fixed time intervals and fixed volumes. Our results showed an increase in the secretion of inflammatory cytokines in airway epithelial cells following sensitization to DFE. Importantly, our study demonstrated that miR-146a-5p-engineered EVs derived from hucMSCs exhibited enhanced anti-inflammatory efficacy in this model.

The NF-κB pathway has long been recognized as a classical proinflammatory signaling pathway (39). For instance, increased expression of NF-κB has been observed in the mucosal cells of patients with inflammatory bowel disease (IBD), while pharmacological interventions targeting NF-κB can ameliorate intestinal inflammation in mouse models of colitis (40). Similarly, studies have demonstrated that inhibition of TLR4/MyD88/NF-κB signaling can attenuate asthma and protect airways against allergic responses and inflammation (41). Additionally, NF-κB regulates the activation of the NLRP3 inflammasome, which results in the production of proinflammatory cytokines such as IL-1β and IL-18 (7, 42). Research by Besnard et al. has revealed that activation of the NLRP3 inflammasome is critical for allergic airway inflammation (43). MiR-146a has been shown to target and reduce the expression of TRAF6 and IRAK1, leading to decreased NF-κB activity (30, 44). Moreover, miR-146a, which depends on the inactivation of the NF-κB pathway, has been found to inhibit the production of pro-inflammatory chemokines in human bronchial epithelial cells (18). Furthermore, other studies have provided evidence that hucMSC-EVs effectively alleviate the activation of the NLRP3 inflammasome, a process regulated by TRAF6, which is inhibited by miR-146a-5p within hucMSC-EVs (45). In our study, we detected changes in associated inflammatory proteins as well as nuclear translocation of NF-κB. Our experimental results initially confirmed that miR-146a-5p-engineered hucMSC-derived EVs attenuate inflammation by regulating NF-κB and NLRP3. This not only partially elucidates the underlying mechanisms, but also offers a novel perspective on improving anti-inflammatory effects by utilizing engineered EVs. However, further exploration of the relevant inflammatory pathways is still warranted.

In conclusion, our study offers a potential therapeutic option for enhancing anti-inflammatory effects in *D. farinae*-induced allergic airway inflammation. The use of modified engineered EVs holds promise for the diagnosis and treatment of various allergic diseases. As suggested by previous research, combining engineered exosomes with classical immunotherapies may provide more beneficial therapeutic benefits (46). Nonetheless, further exploration and clinical investigations will be necessary to fully realize the clinical applications of these findings in the future.

## Data Availability

The raw data supporting the conclusions of this article will be made available by the authors, without undue reservation.
